# Argument mining as rapid screening tool of COVID-19 literature quality: Preliminary evidence

**DOI:** 10.3389/fpubh.2022.945181

**Published:** 2022-07-18

**Authors:** Gianfranco Brambilla, Antonella Rosi, Francesco Antici, Andrea Galassi, Daniele Giansanti, Fabio Magurano, Federico Ruggeri, Paolo Torroni, Evaristo Cisbani, Marco Lippi

**Affiliations:** ^1^Istituto Superiore di Sanità, Rome, Italy; ^2^Department of Computer Science and Engineering, University of Bologna, Bologna, Italy; ^3^Department of Sciences and Methods for Engineering, University of Modena and Reggio Emilia, Reggio Emilia, Italy

**Keywords:** COVID-19, artificial intelligence, argument mining, scientific literature quality assessment, inter-rater agreement

## Abstract

**Background:**

The COVID-19 pandemic prompted the scientific community to share timely evidence, also in the form of pre-printed papers, not peer reviewed yet.

**Purpose:**

To develop an artificial intelligence system for the analysis of the scientific literature by leveraging on recent developments in the field of Argument Mining.

**Methodology:**

Scientific quality criteria were borrowed from two selected Cochrane systematic reviews. Four independent reviewers gave a blind evaluation on a 1–5 scale to 40 papers for each review. These scores were matched with the automatic analysis performed by an AM system named MARGOT, which detected claims and supporting evidence for the cited papers. Outcomes were evaluated with inter-rater indices (Cohen's Kappa, Krippendorff's Alpha, s^*^ statistics).

**Results:**

MARGOT performs differently on the two selected Cochrane reviews: the inter-rater indices show a fair-to-moderate agreement of the most relevant MARGOT metrics both with Cochrane and the skilled interval scores, with larger values for one of the two reviews.

**Discussion and conclusions:**

The noted discrepancy could rely on a limitation of the MARGOT system that can be improved; yet, the level of agreement between human reviewers also suggests a different complexity between the two reviews in debating controversial arguments. These preliminary results encourage to expand and deepen the investigation to other topics and a larger number of highly specialized reviewers, to reduce uncertainty in the evaluation process, thus supporting the retraining of AM systems.

## 1. Introduction

The COVID-19 disease impacted the world in unprecedented ways, prompting a huge effort within the scientific community toward understanding COVID-19 and developing countermeasures to face the emergency. The dramatic spread of the pandemics showed once more that efficient and effective medical treatments and appropriate healthcare responses strongly depend on the coordination, collaboration, and circulation of information within the scientific community (1). A crucial enabling factor turned out to be the ability to rely on timely, evidence-based and unbiased syntheses of available scientific and public health data. For this reason, at the onset of the COVID-19 emergency, the Italian National Institute for Health (Istituto Superiore di Sanità, ISS) set up a working group to review more than 1,000 scientific papers (articles, editorial letters, communications and reviews) related to COVID-19 (2). However, the sheer amount of research papers produced in a relatively short time span—literally thousands of new studies being published each week on COVID-19 (3)—brought the additional challenge of processing all the potentially useful information. A further consideration when approaching COVID-19 related manuscripts is the large number of preprint articles on data repositories. Preprints are not peer-reviewed. Although often rushed to post and quality-wise highly heterogeneous (4, 5), such manuscripts facilitate the rapid dissemination of findings and are particularly suited to support efforts in understanding the disease in real-time as the outbreak unfolds and finding timely solutions. As of June 15, 2021, more than 140,000 manuscripts on COVID-19 had been published or posted as preprints at PubMed, BioRxiv, and MedRxiv on COVID-19 from researchers from all countries.

This situation motivated more than ever the need for reliable tools to automatically sift through overwhelmingly large collections of unstructured, textual data and help experts quickly identify the relevant pieces of information. Recognizing such a need, large datasets of scientific papers have been released (6), challenges have been launched^1^ and tools are being developed to efficiently mine COVID-19 literature with artificial intelligence (7).

In the present paper, we address the challenge of developing automated tools for mining scientific literature related to COVID-19 in particular, and to medicine and healthcare in general, by leveraging on recent advances in argument mining (AM) (8). This is a rapidly expanding research area and technology (9) which seems particularly suitable for the analysis of scientific literature in medicine, although it was never used for this purpose. In particular, AM is an area of natural language processing aimed at extracting arguments from text written in a spoken language, such as English. Argument consist of a statements (usually called claims) about a certain area of interest, often accompanied by supporting evidence. AM tools such as MARGOT (10) have been used for the automated analysis of clinical trials (11) and Amazon reviews (12).

Hence, our study started with the development of a retrieval and ranking tool based on MARGOT, called AMICA (13).^2^ AMICA can automatically process scientific articles, and identify features that are relevant to a key phrase given in input (user query), for example, a sequence of keywords linked to a particular pathology. AMICA uses such features to retrieve relevant papers, and computes various rankings based on the output of MARGOT.

**Figure 1:**
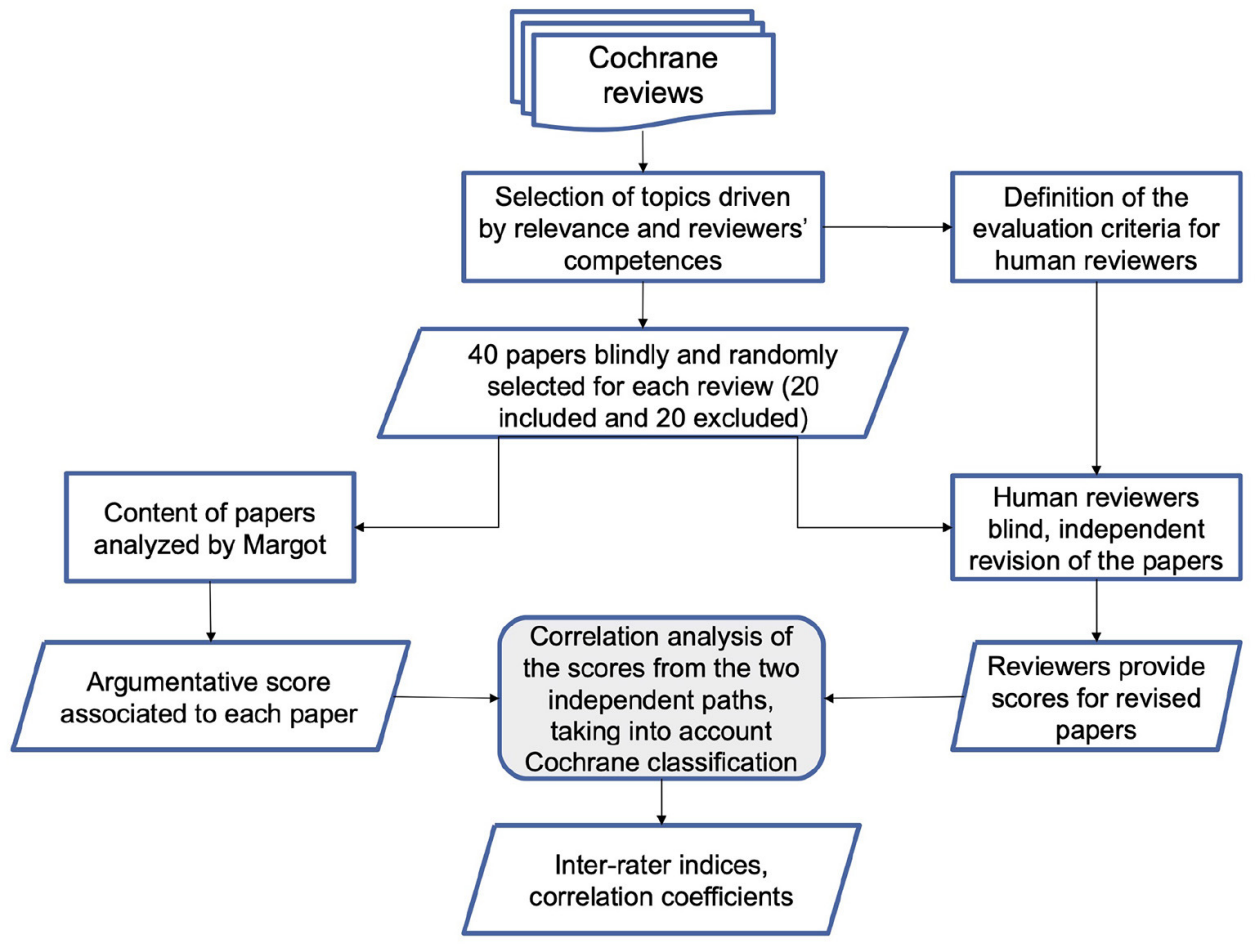
Flowchart representing the methodology adopted to compare MARGOT and human reviewers.

The main goal of the paper is a focus on the methodology and the analysis of the preliminary performance of the AMICA system. To this purpose, we compare the AMICA results with the evaluations of the ISS working group members, on the basis of a predefined validation and test protocol. The protocol includes a classification of the relevance of claims and evidence proposed by the AM system, together with an independent blind classification of the ISS researchers on a list of 40 papers (20 included and 20 excluded) considered in two Cochrane reviews (Figure 1). As a final note, we shall remark that COVID-19 is the case study that motivated the development of AMICA, but the methodology we present can as well be applied to different domains of bio-medicine, health and wellbeing.

## 2. Methodology

This section illustrate the AM engine at the core of the AMICA system and its application to the automatic analysis of Cochrane Systematic Reviews (CSR).

### 2.1. MARGOT

In argumentation theory (14), claims are typically statements or assertions regarding a certain topic, whereas evidence are pieces of information that usually support claims. MARGOT exploits machine learning techniques to detect claims and evidence. It was trained to recognize such argument components using a large corpus developed by IBM in the context of the Debater project.^3^

MARGOT first performs a segmentation of the input text into sentences (typically, separated by a period). Then, for each sentence, MARGOT produces two independent scores: a claim score (CS) and an evidence score (ES). These scores quantify MARGOT's confidence that the sentence contains a claim or a piece of evidence. They are real numbers. A positive claim or evidence score indicates that the sentence is predicted to contain a claim (respectively, an evidence). The higher the score, the more confident is MARGOT that the sentence contains that argument component. By aggregating these sentence-wise scores, one can compute document-level scores that measure the amount of argumentative content that is detected by MARGOT. In our experimental evaluation we computed the following indicators for each document (article):

- **CR**: the percentage (ratio) of sentences containing a claim;
- **ER**: the percentage (ratio) of sentences containing evidence;
- **AR**: the percentage (ratio) of sentences containing either a claim or an evidence;
- **ACS**: the average claim score over all the document's sentences;
- **AES**: the average evidence score over all the document's sentences;
- **AAS**: the average argumentative score, defined as the average highest score (between CS and ES) over all the sentences in the document;
- **PERC**: the 10-th percentile of argumentative scores.

### 2.2. Cochrane systematic reviews

For our experiments, we selected two Cochrane systematic reviews on two rather different topics. One review was about the rapid antigen and molecular-based diagnostic tests for SARS-CoV-2 infection (15). The other was about the thoracic imaging tests for early diagnosis of COVID-19 (16). Both reviews show the last update on September 30th, 2020. Cochrane inclusion criteria for both reviews are reported in Section A of the Supplementary Material.

### 2.3. MARGOT score vs. cochrane criteria and expert's score

For each of the two reviews, we randomly selected 40 papers and assigned them blindly to the reviewers. The choice of papers was performed to get a statistically significant number of papers to be analyzed in a blind way as concerns their inclusion (*N* = 20) or exclusion (*N* = 20) position in the Cochrane reviews, without any other quantitative evaluation or ranking of the papers. This sample size was considered reasonable for a pilot study and appropriate with respect to the overall paper set size: the overall number of included/excluded papers in the two Cochrane reviews is in fact 78/135 and 51/42 for Cochrane 1 and 2, respectively. We also remark that the papers considered in the Cochrane reviews are typically peer-reviewed, with the exception of few pre-prints that have sometimes been included during the pandemics.

The 40 papers were also processed both by MARGOT and by a pool of multidisciplinary experts (researchers in public health, biology, physics, medical science, 2 with laboratory and 2 with imaging diagnostics expertise on COVID-19 literature). We considered two settings. In the first one, MARGOT's indicators, described in the previous paragraph, were compared with the inclusion/exclusion criteria of the corresponding Cochrane systematic review. In the second setting, the same indicators were compared with the blind grading score provided by the experts. The overall methodology is illustrated in the flowchart of Figure 1.

The group of experts graded the 80 papers (40 from each Cochrane review) as independent readers by referring to a set of indicators, illustrated in Section B of the Supplementary Material.

The scores provided by the reviewers (either humans or MARGOT) are numerical fractional or continuous data, whose levels of agreement were evaluated by different inter-rater indices, after interval categorization: the traditional weighted Cohen's Kappa statistics, with Fleiss-Cohen weights (17) applied to all combinations of two-raters; the modified Fleiss Kappa s^*^ weighted statistics (18) expected to mitigate the paradoxical behaviors of the traditional Kappa indices; the Krippendorff's Alpha, designed to apply to various scales of data including ordinal and interval, which can work with two or more raters and is robust to missing data (reviews). In addition, the level of consistency has also been evaluated by the Spearman's rank correlation coefficient. We did not use IntraClass Correlation Coefficients, which are more suitable for continuous scores, since in their standard application they require either (i) each paper to be rated by all reviewers or (ii) the raters to be randomly drawn from a larger population of raters (19, 20). In fact, due to specific expertise of the human raters and their available time, individual papers have been reviewed by one or more human reviewers (two-way random effect models not applicable) depending on their expertise and not randomly (one-way random effects model is also not applicable).

## 3. Results

Following the methodology described in the previous section, we considered 40 papers analyzed by Dinnes et al. and 40 papers by Islam et al.'s reviews and extracted the argument components with the MARGOT tool. Half of the documents were included in the Cochrane, and half were excluded from it.

For each paper, we collected a set of statistics representing the amount of argumentative content detected by MARGOT as described in the Section 2. They are summarized in Supplementary Table 1.

**Figure 2:**
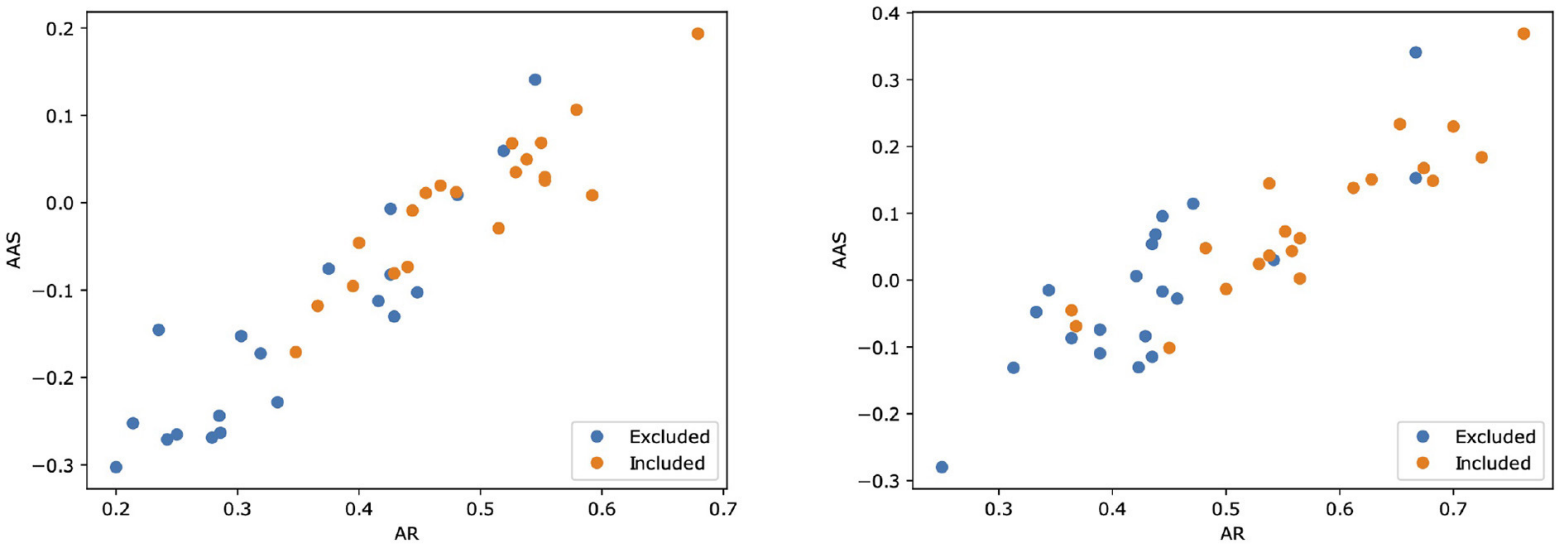
Scatter plots illustrating how two scores computed by MARGOT (namely, AR and AAS) well relate to the eligibility criteria of the two Cochrane reviews. **Left**: review #1; **right**: review #2. Orange (respectively, blue) dots correspond to papers that are included (respectively, excluded) in the review.

Then, we assessed the alignment between MARGOT's indicators and the paper's inclusion in the Cochrane systematic review. Figure 2 shows scatter plots for both Reviews where two indices extracted by MARGOT (namely, AR and AAS) are related with the inclusion/exclusion criteria. Included papers are represented by orange circles, whereas excluded papers are represented by blue ones. A clear correlation can be observed between inclusion in the Cochrane and high argumentative scores (top-right corner in the scatter plot).

**Figure 3:**
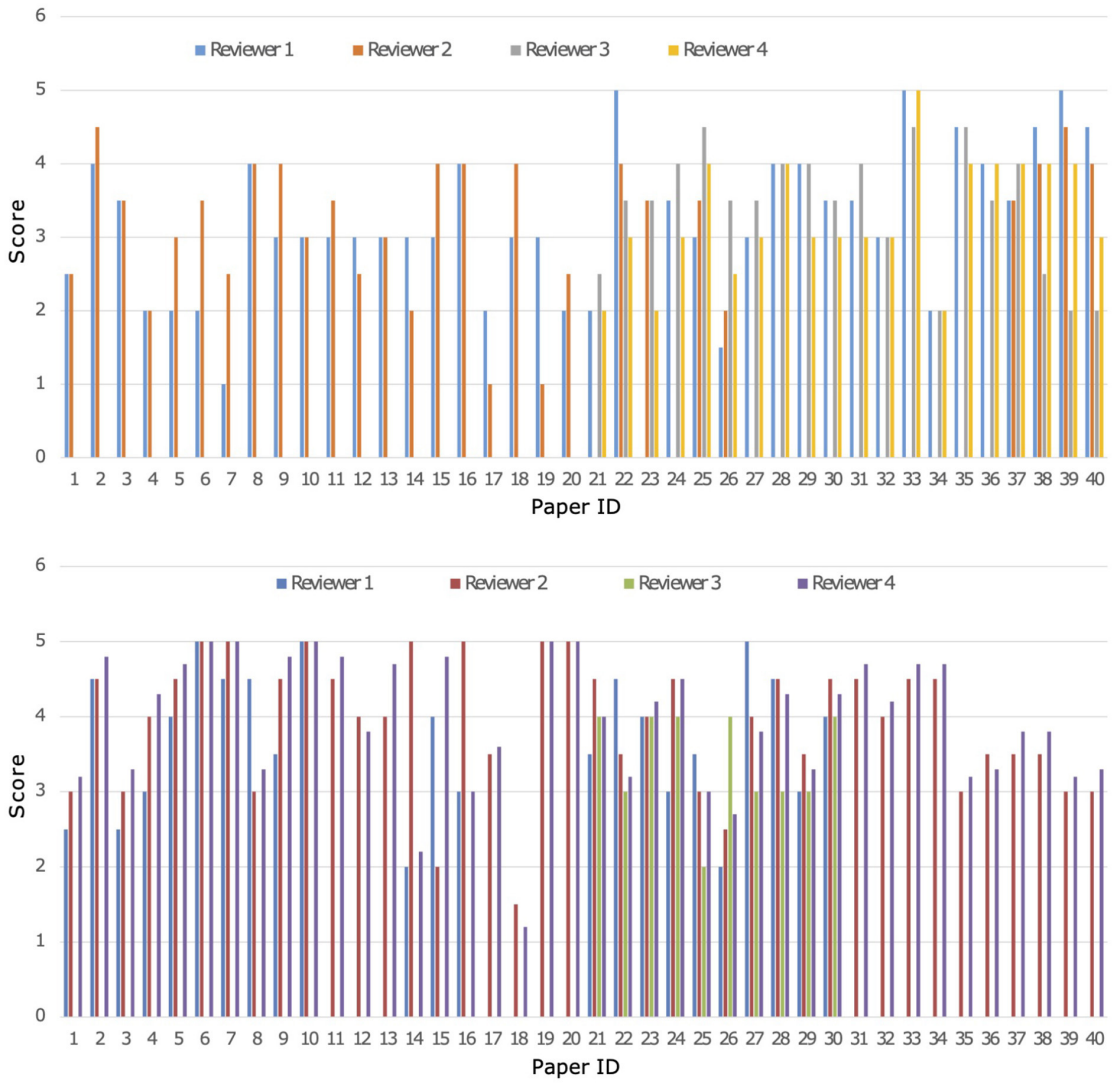
Comparison of grading from the four experts named 1-4 on the 40 papers of Cochrane 1 **(top)** and Cochrane 2 **(bottom)** reviews, Score ranges from 1 to 5.

A parallel and blind evaluation analysis was conducted by the four experts on the 40 papers collected from both Cochrane reviews. As evaluation criteria, the experts referred to a set of quality indicators reported in Appendix B in the Supplementary Material. On the basis of the different reviewer's expertise all 40 papers were revised by 2 out of the 4 researchers for each Cochrane review, respectively, while the others 2 revised 20 papers. A crossed grading was performed for papers with a high score divergence. Both MARGOT and experts were unaware of the state of inclusion or exclusion of the 40 papers. Figure 3 shows a comparison among the results of the expert grading for Cochrane 1 and 2.

**Figure 4:**
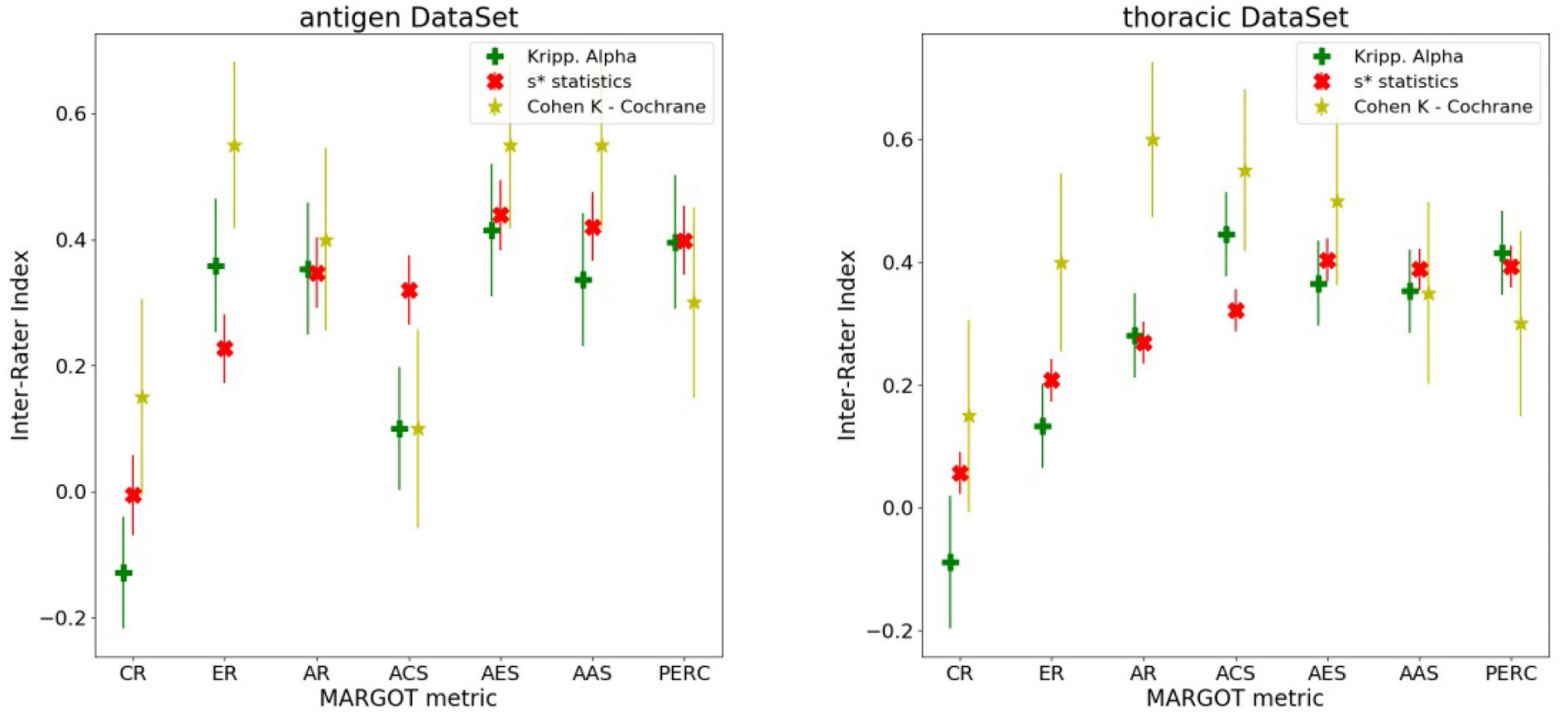
Different inter-rater indices vs. the MARGOT metrics, described in the text (8 categories considered). The yellow stars correspond to the Cohen Kappa index between MARGOT score and Cochrane binary accepted/not-accepted. The error bars represent the Standard Deviations, which are evaluated according to (21) for the Cohen Kappa, and as Standard Deviation of the null hypothesis (agreement due to chance) for the Krippendorff Alpha and s^*^ statistics as described in (18).

Each MARGOT indicator was compared to the human scores, evaluating the multi-rater Alpha and Kappa agreement indices introduced above. To assess the level of consistency, Spearman's correlation was computed between each MARGOT metric and the mean of the human raters. Finally, the comparison between MARGOT scores vs. Cochrane acceptance has been evaluated by the Cohen's Kappa index. The results obtained for both reviews are summarized in Figure 4, whereas additional details on the Spearman's rank correlation coefficient are reported in Supplementary Tables 1, 2.

For most of the MARGOT metrics the reliability is fair-to-moderate according to the Kappa scale interpretation in (22). The Spearman factors show moderate correlations (with consistently small *p*-values) for all metrics except CR and ACS. According to the s^*^ statistics and Krippendorff's Alpha indices combined to Spearman's factor, the AR, AES, AAS, and ER offer the most stable and more than 4 standard deviations away from null hypothesis (agreement due to chance only).

The agreement and consistency between humans and AAS MARGOT have been further investigated by the 2-raters indices evaluated on all possible permutations of two reviewers; this provides further information on how the MARGOT rating is different from humans. The results are summarized in Supplementary Figure 1 (Supplementary Material), which reports the classical Cohen's Kappa together with the Krippendorff's Alpha and s^*^ statistics (left plot) and the Spearman's coefficient between each rater (right plot). These plots confirm the slight-to-moderate agreement (Landis and Koch scale) and similar consistency also between human raters, which is not unusual in reviewing (23, 24); MARGOT metric does not show a relevant difference respect to the human reviewers for the Cohen and Krippendorff indices, and consistency is similar to Cohen reliability; the s^*^ statistics is more sensitive to the presence of MARGOT.

Figure 4 also indicates that the performance of MARGOT is worse on the second case study. This is confirmed by the low or even negative Spearman's coefficients (Supplementary Tables 1, 2) whose large *p*-values tend to support random consistency. However, the multi-raters Alpha and s^*^ statistics indices present similar values, tendentially lower but more correlated than in the first case study and statistically different from the null hypothesis: at least 3 standard deviations away from null hypothesis for AR, AES and AAS and PERC; the Cohen's Kappa to Cochrane review evaluation is generally lower but still significantly different from 0 (more than 2 standard deviations for AR, AES and AAS). Supplementary Tables 2, 3 report the same results in tabular form.

## 4. Discussion

Peer reviewing is a central process in modern research and essential for ensuring high quality and reliability of published work. At the same time, it is a time-consuming process and increasing interest in emerging fields often results in a high review workload, especially for senior researchers in this area. How to cope with this problem is an open question and a subject of intense debate. Tools based on AI may provide assistance to editors, meta-reviewers, and reviewers. Because the decision process in the field of scientific publications is driven by arguments, automatic argument identification methods could provide useful indicators. Importantly, such indicators would be interpretable, since the extracted arguments can be highlighted in a review without detaching them from their context.

The peer-reviewing process of manuscripts and scientific proposals famously suffers from a variability among reviewers' scores according to the different subjects and the presence/absence of a shared evaluation grid (25). This idea drove our attention to the choice of Cochrane Systematic Reviews (CSRs) as a reference point for publications with certain quality standards. CSRs, in fact, aim to identify, appraise and synthesize all the empirical evidence that meets pre-specified eligibility criteria to answer a specific research question. Each CSR performs a thorough screening of the scientific literature related to a given topic (in our case, related to COVID-19) listing those papers that meet, or do not meet, the set of pre-defined eligibility criteria. For this reason, CSRs were chosen as benchmark to test the performance of argument mining technologies, as the rigor of their methods is widely acknowledged, and they are periodically updated in light of new evidence. Moreover, researchers conducting systematic reviews use explicit criteria to minimize bias and produce reliable findings to inform decision-makers. This kind of approach, based on a pre-review agreement of the qualifying points of a manuscript for its inclusion/exclusion, proved to reduce evaluation disparity (26).

Owing to the above, it seemed appropriate in this work to start from inclusion and exclusion criteria used to select papers from two different Cochrane reviews and to check the evaluation agreement between the score proposed *via* argument mining and by internal reviewers. A recent paper explored the use of AI (RobotRewiever) in the evaluation of Randomized Control Trials included in nursing-related Cochrane reviews, leading to a moderate degree of agreement with human reviewers, and suggesting a human supervision of the semi-automated assessment process (27). Nevertheless, it is worth noting that the fitness of the manuscript with the Index Case criteria set in Cochrane reviews is not always correlated with the overall quality of the paper, but rather with the answering to the target questions posed by the stakeholders.

The empirical analysis conducted on the two CSRs gave promising results, although with interesting and remarkable differences between the two case studies. First of all, it is evident that the argumentative scores computed by MARGOT well correlate with the Cochrane inclusion/exclusion criteria. Moreover, MARGOT performs much better in the case study on antigen rapid tests than in that on thoracic imaging. In particular, the 2-rater comparison reported in Supplementary Figure 1 confirms that MARGOT performs worse than human reviewers. However, it also shows a larger variation of agreement and consistency (from poor to substantial according to Landis and Koch) between human raters, which possibly hints that the thoracic dataset is related to a more controversial topic.^4^ This result may suggest that different topics, and thus different research questions, are elaborated and discussed by authors with different argumentative structures.

The preliminary study presented in this paper clearly presents some limitations, which will be addressed in future work. First of all, a wider experimental study should be conducted, involving a larger number of reviewers and covering a variety of heterogeneous topics [e.g., also including COVID-19 readmission and risk factors (28)]. This would enable a more comprehensive analysis of the differences in agreements amongst human reviewers, and between humans and AI/AM tools. Moreover, in order to obtain more homogeneous scores from human reviewers, a set of guidelines associated to each topic will be provided to the researchers involved in the study. Finally, different AM tools will be tested, to assess the impact of different machine learning technologies on the overall methodology: within this context, a more challenging research direction would be to use annotations provided by experts to train a new AM system.

## Data availability statement

The original contributions presented in the study are included in the article/Supplementary Material, further inquiries can be directed to the corresponding author/s.

## Author contributions

FA, AG, and FR organized the databases used in the experimental evaluation. GB, AR, DG, and FM performed the scoring of papers. EC, ML, and PT performed the statistical analysis. GB, AR, EC, ML, and PT wrote the sections of the manuscript. All authors contributed to the conception design of the study and to manuscript revision, read, and approved the submitted version.

## Funding

The research conducted in this paper was supported by the Italian Ministry for Education and Research, under the FISR-COVID 2020 project AMICA.

## Conflict of interest

The authors declare that the research was conducted in the absence of any commercial or financial relationships that could be construed as a potential conflict of interest.

## Publisher's note

All claims expressed in this article are solely those of the authors and do not necessarily represent those of their affiliated organizations, or those of the publisher, the editors and the reviewers. Any product that may be evaluated in this article, or claim that may be made by its manufacturer, is not guaranteed or endorsed by the publisher.

## Footnotes

^1^ https://www.kaggle.com/covid-19-contributions

^2^ http://amica.unimore.it/

^3^ https://research.ibm.com/interactive/project-debater/

^4^We argue that this kind of behavior might be mainly due to the justification principle compliance for medical exposure and also to the more rigorous technical requirements of CT used in thoracic imaging.
