# Supplementary material for: Argument mining as rapid screening tool of COVID-19 literature quality: Preliminary evidence
Source: Front Public Health. 2022 Jul 18;10:945181. doi: 10.3389/fpubh.2022.945181 (PMC9339778; doi:10.3389/fpubh.2022.945181)
Supplement: Supplementary file 1 [file Data_Sheet_1.PDF]

A INCLUSION CRITERIA FOR COCHRANE CASE STUDIES

Cochrane #1: antigen and rapid tests

- Study design: control vs infected groups, and related clinical profiling (asyntomatic + syntomatic and convalescent) and size.
- Accuracy (sensitivity and specificity) for each group considered via confirmation of screened positive and negative samples by real time polymerase chain reaction (RT-PCR)
- Point-of-care practicability (i.e. minimal biosafety requirements, minimal equipments, rapid response)

Cochrane #2: thoracic imaging

- Study design chest CT, chest X-ray, or ultrasound of thelungs, meeting the criteria described in the Index test(s) section.
- The roles of the test could have been a replacement of RT-PCR, an add-on test, a triage test, rapid testing, or used concurrently with other diagnostic tests.
- Only index tests interpreted by humans, and not an algorithm (machine learning/artificial intelligence (AI)).
- Case control studies excluded

B INDICATORS USED FOR PAPER SCORING BY REVIEWERS

Cochrane #1: antigen and rapid tests

- Index test: portable or mains powered device; mimimal sample preparation requirements; minimal biosafety requirements; no requirement for a temperature controlled requirements; test results available with 2 hours of sample collection.
- Reference standard for COVID-19 cases: positive RT-PCR alone or clinical diagnosis of COVID-19 based on established guidelines or combinations of clinical features.
- Reference standard for no COVID-19 cases: negative RT-PCR or pre-pandemic sources of samples.
- Accuracy (Sensitivity/specificity): lack of information
- Conflict of interest: declared or undeclared

Cochrane #2: thoracic imaging

- Accuracy (Sensitivity/specificity): adequate sampling and representativeness for sensitivity and specificity.
- Patient data: data relating to the clinical status of patients.
- Lesion’s picture: data relating to the pathognonomic score of the lung lesions observed.
- RT-PCR and instrumental data correlation: data relating to the time elapsed between RT-PCR and instrumental examination (cause-effect relationship).
- Patient parameters: characterization of subjects for symptom onset versus hospitalization data (false negative RT-PCR).
- Evaluation: comparison between operators of instrumental findings.
- Uncertainties: correct evaluation of the uncertainties related to the study design, in light of the temporal cause-effect relationships.

C ADDITIONAL DETAILS ON EXPERIMENTATION

Table S1. Spearman's rank correlation coefficient between MARGOT metrics and mean core of human raters for Cochrane review #1.

MARGOT score	Spearman's correlation coefficient	p-value
CR	0.111	0.436
ER	0.565	0.00036
AR	0.526	0.00090
ACS	-0.138	0.489
AES	0.508	0.0017
AAS	0.463	0.0045
PERC	0.457	0.0021

Table S2. Spearman's rank correlation coefficient between MARGOT metrics and mean core of human raters for Cochrane review #2.

MARGOT score	Spearman's correlation coefficient	p-value
CR	0.044	0.84
ER	-0.128	0.53
AR	-0.002	0.95
ACS	0.311	0.074
AES	-0.087	0.48
AAS	0.019	0.97
PERC	0.151	0.35

Table S3. Different inter-rater indices versus the MARGOT metrics, for Cochrane #1.

MARGOT score	Krippendorff's Alpha	s*	Cohen's Kappa
CR	-0.13 ± 0.09	-0.01 ± 0.06	0.15 ± 0.16
ER	0.36 ± 0.11	0.23 ± 0.05	0.55 ± 0.13
AR	0.35 ± 0.10	0.35 ± 0.06	0.40 ± 0.14
ACS	0.10 ± 0.10	0.32 ± 0.06	0.10 ± 0.16
AES	0.42 ± 0.11	0.44 ± 0.06	0.55 ± 0.13
AAS	0.34 ± 0.11	0.42 ± 0.05	0.55 ± 0.13
PERC	0.40 ± 0.11	0.40 ± 0.05	0.30 ± 0.15

Table S4. Different inter-rater indices versus the MARGOT metrics, for Cochrane #2.

MARGOT score	Krippendorff's Alpha	s*	Cohen's Kappa
CR	-0.09 ± 0.11	0.06 ± 0.03	0.15 ± 0.16
ER	0.13 ± 0.07	0.21 ± 0.03	0.40 ± 0.14
AR	0.28 ± 0.07	0.27 ± 0.03	0.60 ± 0.13
ACS	0.45 ± 0.07	0.32 ± 0.03	0.55 ± 0.13
AES	0.37 ± 0.07	0.40 ± 0.03	0.50 ± 0.14
AAS	0.35 ± 0.07	0.39 ± 0.03	0.35 ± 0.15
PERC	0.42 ± 0.07	0.39 ± 0.03	0.30 ± 0.15

Figure S1. The classical Cohen's Kappa 2-rater index (left) for all 2-rater permutations compared to multi-rater Krippendorff's Alpha and the s* statistics; the corresponding Spearman's correlation coefficients are reported on the right plot. Top: Cochrane #1; bottom: Cochrane #2. The "plus" and "cross" markers represent the Alpha and s for all raters except the one on the corresponding abscissa. 8 scoring categories have been considered. Rater number 4 corresponds to AAS MARGOT metric. For clarity, standard deviations and p-values are not reported on the plot, they are very similar to those presented in Figure 3 in the main paper and in Table S2, respectively.

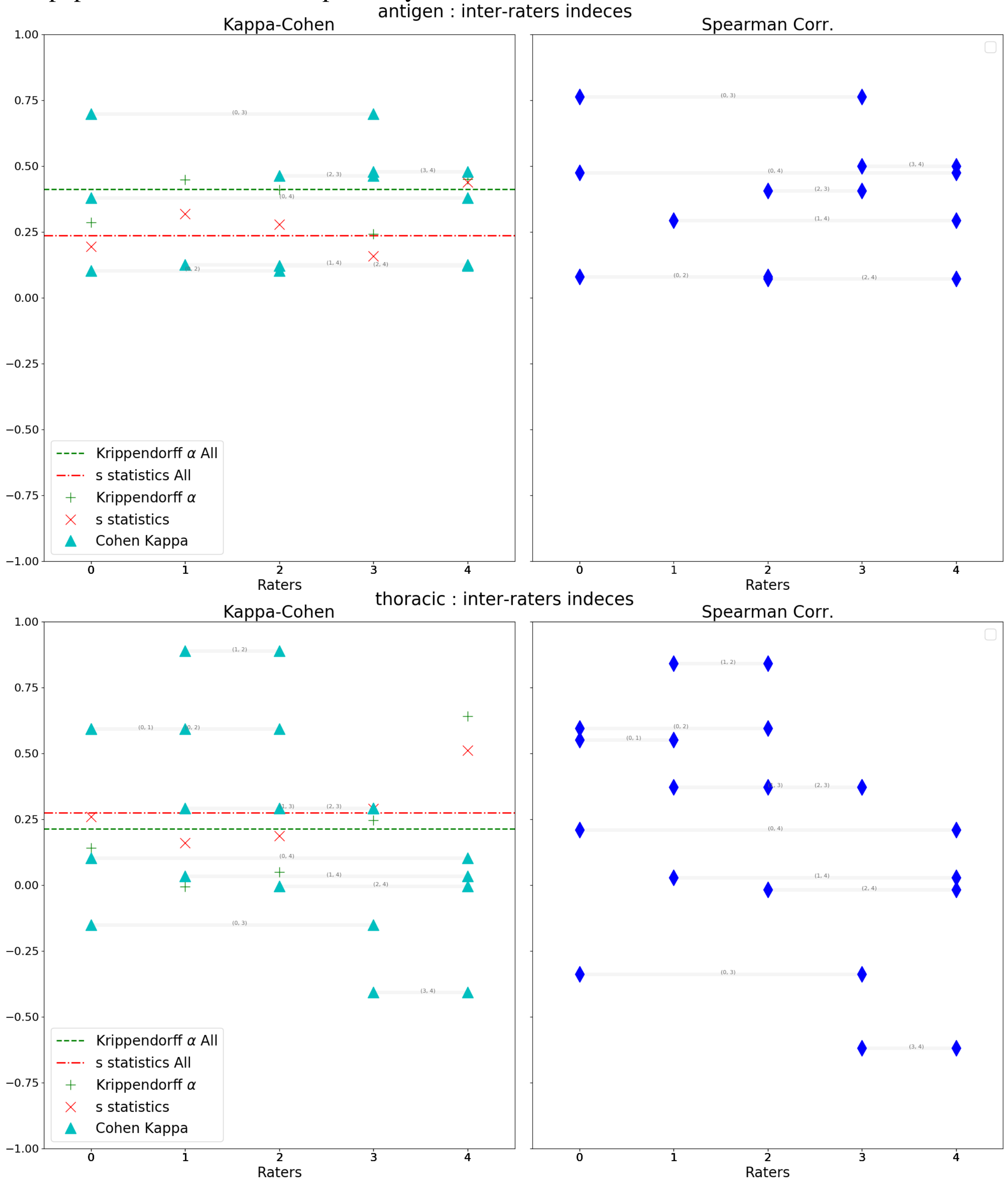

Table S5. List of 40 papers used in our experimental evaluation, taken from Cochrane #1 (Dinnes et al., 2021). Left: included papers; right: excluded papers. We adopted the same naming used in the list provided by (Dinnes et al., 2021)).

Albert 2020	Ai 2020
Alemany 2020	Anahtar 2020
Assennato 2020	Ar Gouilh 2020
Blaidon 2020	Arizti-Sanz 2020
Broder 2020	Arumugam 2020
Cerutti 2020	Avetyan 2020
Chen 2020a	Azhar 2020
Courtellemont 2020	Azzi 2020
Cradic 2020	Baek 2020
Diao 2020	Barra 2020
Dust 2020	Basu 2020
Ghofrani 2020	Behrmann 2020
Gibani 2020	Bokelmann 2020
Goldenberger 2020	Bordi 2020
Gremmels 2020a	Brandsma 2020
Hogan 2020	Broughton 2020
Hou 2020	Bull 2020
Jokela 2020	Bulterys 2020
Kruger 2020	Callahan 2020
Lambert-Niclot 2020	Chandler-Brown 2020

Table S6. List of 40 papers used in our experimental evaluation, taken from Cochrane #2 (Islam et al., 2021). Left: included papers; right: excluded papers. We adopted the same naming used in the list provided by (Islam et al., 2021)

Ai 2020a	Ai 2020b
Aslan 2020	Ai 2020c 2020
Bar 2020 2020	Arentz 2020
Bellini 2020	Bai 2020a
Besutti 2020	Bai 2020b
Borakati 2020 2020	Chang 2020
Cartocci 2020	Chen 2020b
Caruso 2020 2020	Chen 2020c
Cozzi 2020	Cheng 2020
Debray 2020	Cinkooglu 2020
De Smet 2020	Colombi 2020
Dini 2020	Dai 2020
Ducray 2020	Ding 2020
Falaschi 2020	Dong 2020
Fonsi 2020a	Himoro 2020
Fujioka 2020	Liang 2020
Gezer 2020	Mao 2020
Giannitto 2020	Miao 2020a
Gietema 2020	Miao 2020b
Guillo 2020	Pakray 2020
